# Examine m.3243A>G carriers prospectively and comprehensively, treat them symptomatically, and avoid mitochondrion-toxic drugs 

**DOI:** 10.5414/CNCS111564

**Published:** 2024-10-30

**Authors:** Josef Finsterer

**Affiliations:** Neurology Department, Neurology & Neurophysiology Center, Vienna, Austria

**Keywords:** mtDNA, m.3243A>G, kidney failure, respiratory chain, maternal transmission

## Abstract

None.

Sir, – We read with interest the article by Kamido et al. [1] about a 16-year-old boy with a mitochondrial disorder (MID) due to the m.3243A>G variant in MT-TL1 (heteroplasmy 48%) who had multisystem disease (brain, ears, heart, kidneys, nerves, lactic acidosis), but most importantly nephropathy. The study is excellent, but some points should be discussed. 

The first point is that we disagree with the statement that the encephalopathy is probably due to rapid dehydration. Encephalopathy is an inherent phenotypic feature in most m.3243A>G carriers. To confirm encephalopathy, it would have been imperative to perform magnetic resonance spectroscopy (MRS) MRS and cerebrospinal fluid (CSF) tests in addition to cerebral MRI and EEG. The MRS can show a reduced NAA peak and an increased lactate peak. CSF exam may reveal increased lactate. 

The second point is that mitochondrial dysfunction was not confirmed by biochemical examination of the kidneys or heart muscle. Since immunohistochemistry and electron microscopy clearly confirmed mitochondrial dysfunction, it would have been crucial to measure the activity of the five respiratory chain complexes. 

Third, the heteroplasmy rate should also have been determined in the kidney and heart muscle, since the kidneys were the most affected organ, and the patient underwent biopsy of these two organs. 

The fourth point is that m.3243A>G carriers often have myopathy, so the patient should also have been tested for muscle disease. 

In summary, carriers of the m.3243A>G variant require a prospective and comprehensive diagnostic work-up, symptomatic treatment, and discontinuation of mitochondrion-toxic drugs. 

## Availability of data and material 

All data are available from the corresponding author. 

## Author’s contributions 

JF was responsible for the design and conception, discussed available data with coauthors, wrote the first draft, and gave final approval. 

## Funding 

None received. 

## Conflict of interest 

The author declare that the research was conducted in the absence of any commercial or financial relationships that could be construed as a potential conflict of interest. 
